# Deoxyribonuclease Is a Potential Counter Regulator of Aberrant Neutrophil Extracellular Traps Formation after Major Trauma

**DOI:** 10.1155/2012/149560

**Published:** 2012-01-23

**Authors:** Wei Meng, Adnana Paunel-Görgülü, Sascha Flohé, Ingo Witte, Michael Schädel-Höpfner, Joachim Windolf, Tim Tobias Lögters

**Affiliations:** Department of Trauma and Hand Surgery, University Hospital Dusseldorf, 40225 Dusseldorf, Germany

## Abstract

*Introduction*. Neutrophil extracellular traps (NET) consist of a DNA scaffold that can be destroyed by Deoxyribonuclease (DNase). Thus DNases are potential prerequisites for natural counter regulation of NETs formation. In the present study, we determined the relationship of NETs and DNase after major trauma. *Methods*. Thirty-nine major trauma patients, 14 with and 25 without sepsis development were enrolled in this prospective study. Levels of cell-free (cf)-DNA/NETs and DNase were quantified daily from admission until day 9 after admission. *Results*. Levels of cf-DNA/NETs in patients who developed sepsis were significantly increased after trauma. In the early septic phase, DNase values in septic patients were significantly increased compared to patients without sepsis (*P* < 0.05). cf-DNA/NETs values correlated to values of DNase in all trauma patients and patients with uneventful recovery (*P* < 0.01) but not in septic patients. Recombinant DNase efficiently degraded NETs released by stimulated neutrophils in a concentration-dependent manner in vitro. *Conclusions*. DNase degrades NETs in a concentration-dependent manner and therefore could have a potential regulatory effect on NET formation in neutrophils. This may inhibit the antibacterial effects of NETs or protect the tissue from autodestruction in inadequate NETs release in septic patients.

## 1. Introduction

Major trauma is associated with the induction of systemic inflammation, development of sepsis, and multiple organ failure (MOF) [1–3]. Neutrophils are the main types of effector cells in the innate immune system and are the first line against infection [4–7]. Activated polymorphonuclear neutrophils play a pivotal role in the systemic inflammatory response syndrome (SIRS) and development of sepsis after major trauma [8].

In addition to the more traditional mechanism of phagocytosis to kill bacteria, it has recently been shown that activation of neutrophils can cause the release of neutrophil extracellular traps (NETs) [9]. NETs are large DNA-associated molecule complexes carrying nucleic and cytoplasmic proteins such as histones, elastase, myeloperoxidase (MPO), pentraxin, lactoferrin, and bactericidal/permeability-increasing protein (BPI) [10, 11]. Each of them has got strong antimicrobial and/or immunomodulating properties. Upon activation (by e.g., IL-8, lipopolysaccharide, bacteria, fungi, or activated platelets) neutrophils start a program that leads to the formation of NETs [12–14]. The formation of NETs, recently termed “NETosis”, is an active process and is distinct from neutrophil apoptosis and necrosis involving *postmortem* antimicrobial and proinflammatory immune responses [15]. NETs provide a high local concentration of antimicrobial components and bind, disarm, and kill microbes extracellularly as an emergency first line defense mechanism [9]. NET trapping in the tissue may allow the host to confine an infection and thus reduce the likelihood for the pathogens to spread into the bloodstream [12]. On the other hand, the high local concentration of NETs-associated effector molecules may contribute to severe tissue damage and organ dysfunction and/or failure [16].

Deoxyribonuclease (DNase) I, a Ca^2+^/Mg^2+^-dependent endonuclease, is the major nuclease found in body fluids such as serum and urine. Its primary function has been assumed to be the degradation of dietary DNA within the alimentary tract. Moreover, it has been shown that extracellular DNase may account for the chromatin breakdown during necrosis as a basis of protection against anti-DNA autoimmunity. DNA is the major structural component of NETs with granule proteins attached to this DNA backbone [9]. Thus, the DNA scaffold of NETs can be destroyed by DNase. So, aberrant NET formation in combination with lack of patient's DNases degrading NETs might contribute to their prolonged persistence with subsequent tissue damage and/or autoimmune diseases [17–20]. Furthermore, DNases are expressed by several bacterial pathogens. Bacterial DNases act as a virulence determinant by counteracting NETs-mediated trapping, thereby promoting bacterial spread from local sites to the bloodstream [17, 18].

Recently it has been shown that NETs kinetics followed the inflammatory course after severe trauma [21]. In the current study we aimed to determined cfDNA/NETs and DNase in the serum of critically ill patients during the early posttraumatic phase. We further provide evidence for a DNase-mediated dissolution of NETs in vitro.

## 2. Materials and Methods

### 2.1. Patients

Thirty-nine patients were enrolled in this prospective study. Study approval was obtained from the Ethics Review Board of the University of Duesseldorf, Germany. Patients with blunt or penetrating multiple injuries who were admitted to our Level I Trauma Center with an Injury Severity Score (ISS) >16, and aged 18 years and older were enrolled in this study. Written informed consent was obtained from all participants or their legal representatives if the patients lacked consciousness. Exclusion criteria were death of the patient on day of admission or within the first two days on ICU and withdrawal of patient consent. In addition, patients with known preexisting immunological disorders or systemic immunosuppressive medication were excluded. The severity of injury was assessed by the ISS, based on the Abbreviated Injury Scale (AIS) [19]. SIRS and sepsis were defined using the criteria outlined 2005 from the International Sepsis Forum [20]. Patients were determined as septic if they fulfilled criteria for systemic inflammatory response syndrome and had a proven source of infection. Systemic inflammatory response syndrome was defined by two or more of the following criteria: temperature >38°C or <36°C; heart rate >90 beats per minute; respiratory rate >20 breaths per minute or arterial carbon dioxide tension (PaCO_2_) <32 mmHg; white blood cell count >12.000 cells/mm^3^ or <4.000 cells/mm^3^, or with >10% immature (band) forms. In order to evaluate organ dysfunction/failure, the Sequential Organ Failure Assessment (SOFA) and Multiple Organ Dysfunction (MOD) score were determined prospectively every day. In addition clinical laboratory data, including red and white blood counts, electrolytes, creatinine, blood urea nitrogen, C-reactive protein (CRP), and liver enzymes were monitored daily. Serum and EDTA blood were collected on admission to the emergency room (ER) and on days 1 to 10 after injury. Samples were centrifuged, immediately frozen, and stored at −80°C until further analysis.

### 2.2. cfDNA/NETs Assay

cfDNA/NETs were quantified using the Quant-iT PicoGreen dsDNA assay (Invitrogen GmbH, Darmstadt, Germany). This assay used to label neutrophil-derived NETs by targeting the NET containing cf-DNA directly within serum has been recently developed [22]. The fluorescence intensity reflects the amounts of DNA and was measured at excitation and emission wavelengths of 485 nm and 530 nm, respectively, in a microplate reader (Victor3, PerkinElmer, Waltham, USA) A standard calibration curve by means of defined calf thymus DNA (Sigma) amounts ranging from 0 to 2 *μ*g/mL has been used in all analyses.

### 2.3. Quantification of Desoxyribonuklease (DNase) by ELISA

Desoxyribonuklease (DNase) levels in serum samples were measured by using ORG 590 DNase Activity Immunometric Enzyme Immunoassay for the Quantitative Determination of DNase Activity (ORGENTEC, Mainz, Germany) according to the manufacturer's instructions. Additionally, the concentration of DNase in human sera was quantified using known concentrations of the standard provided with rh-DNase1 (0.75 up to 12.5 ng/mL).

### 2.4. Isolation of Human Neutrophils

Human neutrophils were isolated by discontinuous density-gradient centrifugation on Percoll (Biochrom, Berlin, Germany) as previously described [23]. After hypotonic lysis to remove contaminating erythrocytes, cells were suspended in phosphate-buffered saline (PBS). Purity and viability were routinely >95% as assessed by forward and side scatter characteristics of FACScan (BD, Heidelberg, Germany) and trypan blue exclusion, respectively.

### 2.5. Stimulation of Neutrophils and NETs Release

Freshly isolated neutrophils from healthy volunteers were resuspended in RPMI 1640 containing 2 mM glutamine supplemented with 100 U/mL penicillin, 100 *μ*g/mL streptomycin, and 10% heat inactivated fetal calf serum to a final concentration of 2 × 10^6^/mL. Cells were stimulated with 5 nM PMA for 3 h at 37°C in a humidified atmosphere containing 5% CO_2_. Then cfDNA/NETs release was quantified in the supernatant. In addition, supernatants were incubated with 0, 0.02, 0.2, 2.0, and 10 *μ*g/mL recombinant DNase (Pulmozyme, Roche, Swisse) for 3 h at 37°C before cfDNA/NETs quantification.

### 2.6. Immunofluorescence Staining of NETs

For immunofluorescence, freshly isolated neutrophils were seeded on poly-D-lysin-coated coverslips, allowed to adhere, and stimulated with 50 nM PMA for 3 h at 37°C. Cells were further fixed with 4% PFA and blocked with 5% NGS, 0.3% Triton X-100 in PBS for 30 min. To stain NETs, samples were incubated with a monoclonal mouse antimyeloperoxidase antibody (1 : 300) and a secondary FITC-conjugated antibody (1 : 200; both Dako, Hamburg, Germany). After staining of DNA with DAPI, specimens were mounted in Dako fluorescent mounting medium (Dako). Neutrophil-derived NET formation was visualized by immunofluorescence microscopy (Axiovert 100, Zeiss, Goettingen, Germany).

### 2.7. Statistical Analyses

To evaluate differences between the study groups, a Kruskal-Wallis test with Dunn's post hoc test was performed. Correlation between numerical values was evaluated by Spearman's rank correlation coefficient (*r*). The Mann-Whitney rank sum test was performed to compare two groups of values at the same time. Analyses were performed using GraphPad Prism software (version 5; GraphPad Software, San Diego, CA, USA). Data were considered to be statistically significant at *P* < 0.05.

## 3. Results

### 3.1. Demographics

The 39 patients (28 male, 11 female) enrolled into the study had an ISS of 38.8 ± 2.6 (mean ± SEM, range 16–75). The mean age was 45.2 ± 3.1 years (range 19–82 years). From all patients, 14 developed sepsis (sepsis group) within 5.8 ± 0.4 days (range 4–8 days) after admission. Infection site of sepsis and microbiological pathogens for each patient are depicted in Table 1. Three patients died posttraumatically after 55.2 ± 23.3 days (range 24–147 days). The mean ICU stay was 16.9 ± 2.4 days (range 2–74 days). The mean age of the 14 patients (3 female, 11 male) who subsequently developed sepsis was 50.3 ± 5.8 years (range, 21–82 years). The mean ISS in this patient group was 45.6 ± 4.9 (range, 16–75). Mean ISS in patients without sepsis was 35.0 ± 2.7 (range, 16–66). The mean ICU stay in the sepsis group was 25.5 ± 3.4 days (range 7–50 days) and in the group without development of a sepsis 12.1 ± 2.9 days (range 2–74 days).

### 3.2. cfDNA/NETs and DNase in Sepsis versus No Sepsis Group

cfDNA/NETs and DNase values were determined from admission to the emergency room (ER) until day 9 after trauma. In order to define normal values, blood samples from 10 healthy volunteers were analyzed for cfDNA/NETs (median, 116.3; interquartile range (IQR), 102.8 to 131.7 ng/mL) and DNAse (median, 3.15; IQR, 2.61 to 3.80 ng/mL).

**Figure 1:**
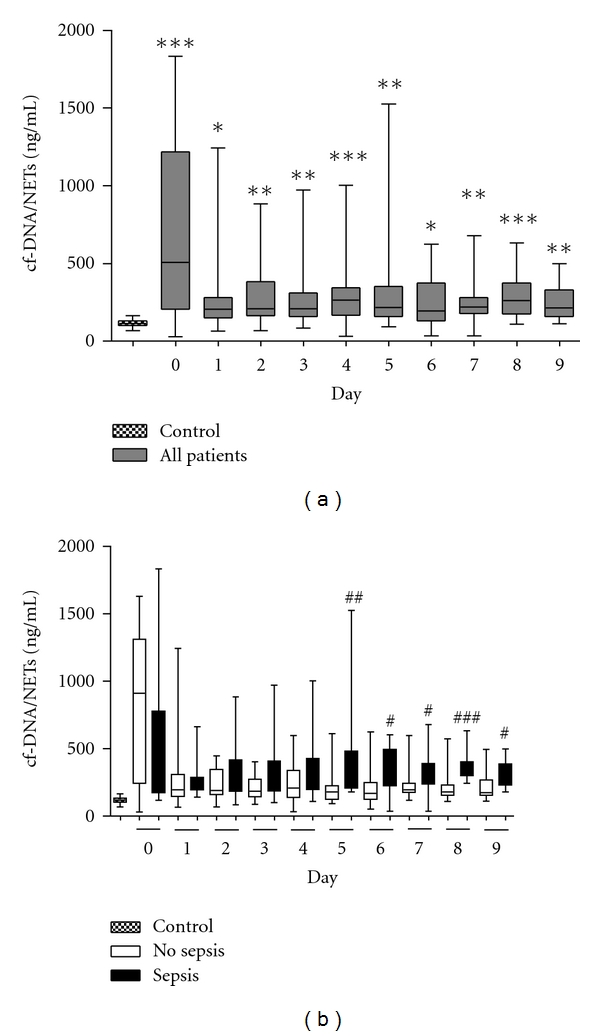
Kinetics of cfDNA/NETs after major trauma. (a) cf-DNA/NETs levels in patients (*n* = 39) were significantly enhanced after trauma (*P* < 0.01), versus control group (healthy volunteers). *P < 0.05, **P < 0.01, and ***P < 0.0001, (b) cfDNA/NETs levels in patients who developed sepsis during the first 10 days after trauma (*n* = 14) were significantly elevated when compared to the values determined in patients with uneventful recovery (*n* = 25). The horizontal line across the boxplots represents the median, and the lower and upper ends of the boxplots are the 25th and 75th percentiles, respectively. Whiskers indicate the minimum and maximum values, respectively versus no sepsis group at the same time. ^#^ *P* < 0.05, ^##^ *P* < 0.01 and ^###^ *P* < 0.0001.

Early after major trauma cf-DNA/NETs values were significantly enhanced in comparison to healthy donors, decreased within the next days but remain elevated above normal values of volunteers for the entire period (Figure 1(a)). However, cfDNA/NETs values of patients who subsequently developed sepsis increased again at day 5 until day 9 after trauma (day 5, median, 334.0; interquartile range (IQR), 209.4 to 482.2 ng/mL), whereas the values for those patients without development of sepsis remained on the same level (day 5, median, 178.3; interquartile range (IQR), 125.8 to 223.4 ng/mL; Figure 1(b)). Significant intergroup differences were detectable between sepsis and nonsepsis patients on days 5 to 9 as shown in Figure 1(b).

**Figure 2:**
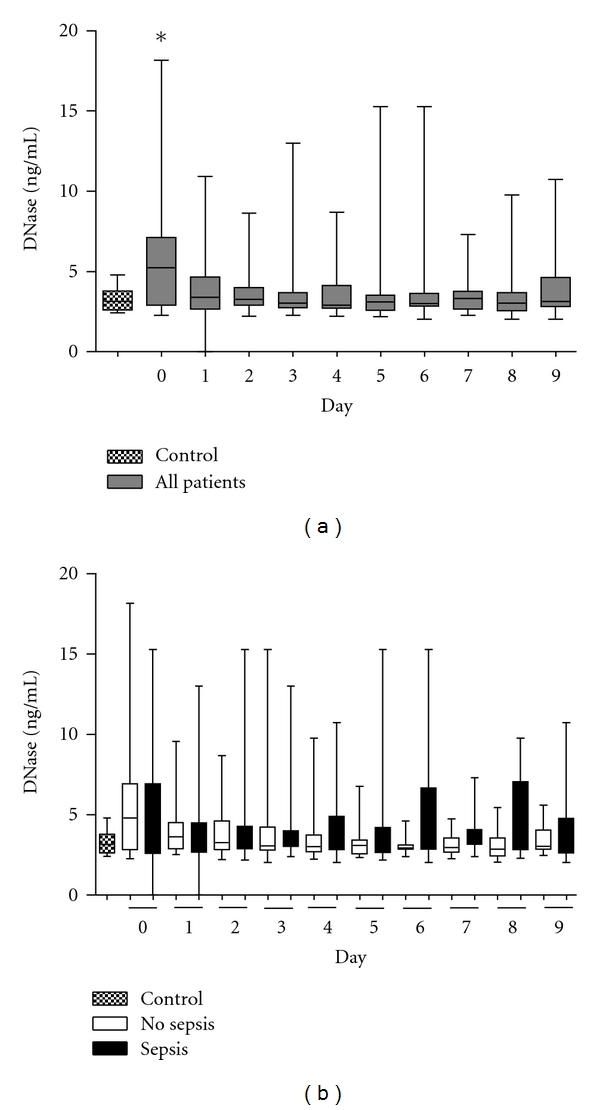
Kinetics of DNase after major trauma. (a) DNase values in patients (*n* = 39) were significantly elevated early after trauma (*P < 0.05 versus control group). (b) DNase levels in patients who developed sepsis during the first 10 days after trauma (*n* = 14) did not show any differences compared to the values determined in patients with uneventful recovery (*n* = 25) or control group (healthy volunteers). The horizontal line across the boxplots represents the median, and the lower and upper ends of the boxplots are the 25th and 75th percentiles, respectively. Whiskers indicate the minimum and maximum values, respectively.

 DNase values were also significantly increased on admission (heathly volunteers: median, 3.145; interquartile range (IQR), 2.614 to 3.799; all patients at day 0: median, 5.715; interquartile range (IQR), 3.400 to 7.119 ng/mL.  Figure 2(a)). Regardless of the development of a sepsis or not, DNase values decreased within the next days for both groups and remained on the control level throughout the entire period.

**Figure 3:**
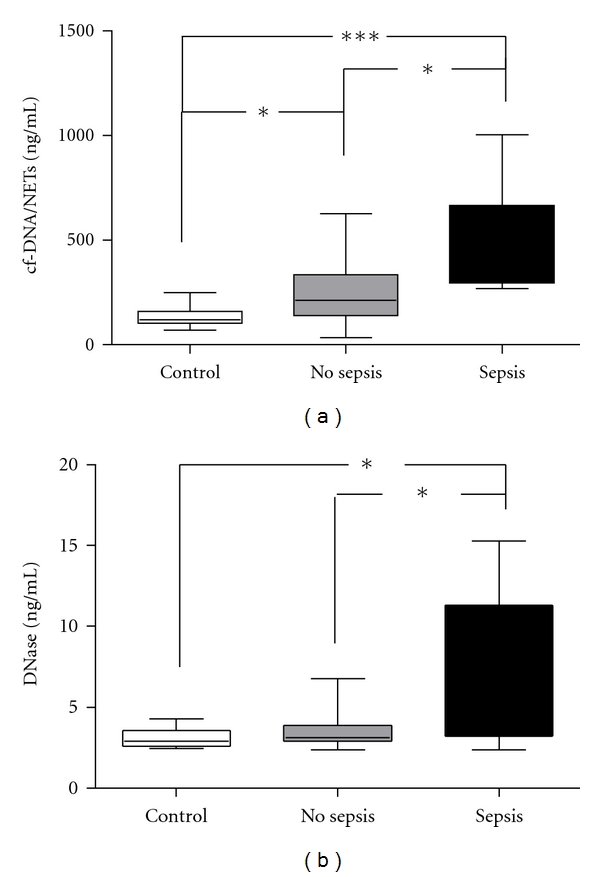
Values of cfDNA/NETs and DNase in the early septic phase. Highest values of cf-DNA/NETs and DNase in the range between the day before (−1), the day of (0), and the day after (+1) diagnosis of sepsis after major trauma. (a) cf-DNA/NETs values of patients with sepsis were significantly enhanced compared to patients with uneventful recovery (*P* < 0.05) or healthy volunteers (*P* < 0.0001). (b) Likewise values of DNase of patients who developed sepsis after major trauma were significantly enhanced compared to patients with uneventful recovery (*P* < 0.05) or healthy volunteers (*P* < 0.05). The horizontal line across the boxplots represents the median, and the lower and upper ends of the boxplots are the 25th and 75th percentiles, respectively. Whiskers indicate the minimum and maximum values, respectively. *P < 0.05 and ***P < 0.0001.

Patients developed sepsis in the mean 5.8 ± 0.4 days (range 4–8 days) after trauma. In order to investigate the potential role of cfDNA/NETs and DNase values as markers for the onset of sepsis, we determined the highest values of both parameters on the day before (−1), the day of (0), and the day after (+1) diagnosis of sepsis (Figure 3). These values were compared to highest values between day 4 and 6 of patients without development of sepsis and healthy volunteers. During the early phase of a sepsis, cf-DNA/NETs and DNase in patients were significantly elevated compared to patients without development of sepsis after major trauma (*P* < 0.05) and healthy volunteers (*P* < 0.0001).

 cf-DNA/NETs values strongly correlate to DNase values (*r* = 0.2523, *P* < 0.0001) after trauma. Furthermore, cf-DNA/NETs concentrations determined in patients without sepsis development after severe trauma strongly correlated with DNase (*r* = 0.207, *P* < 0.01). However, values did not correlate in patients who subsequently developed sepsis (*r* = 0.4771, *P* > 0.05).

### 3.3. Degradation of NETs by DNase Treatment

**Figure 4:**
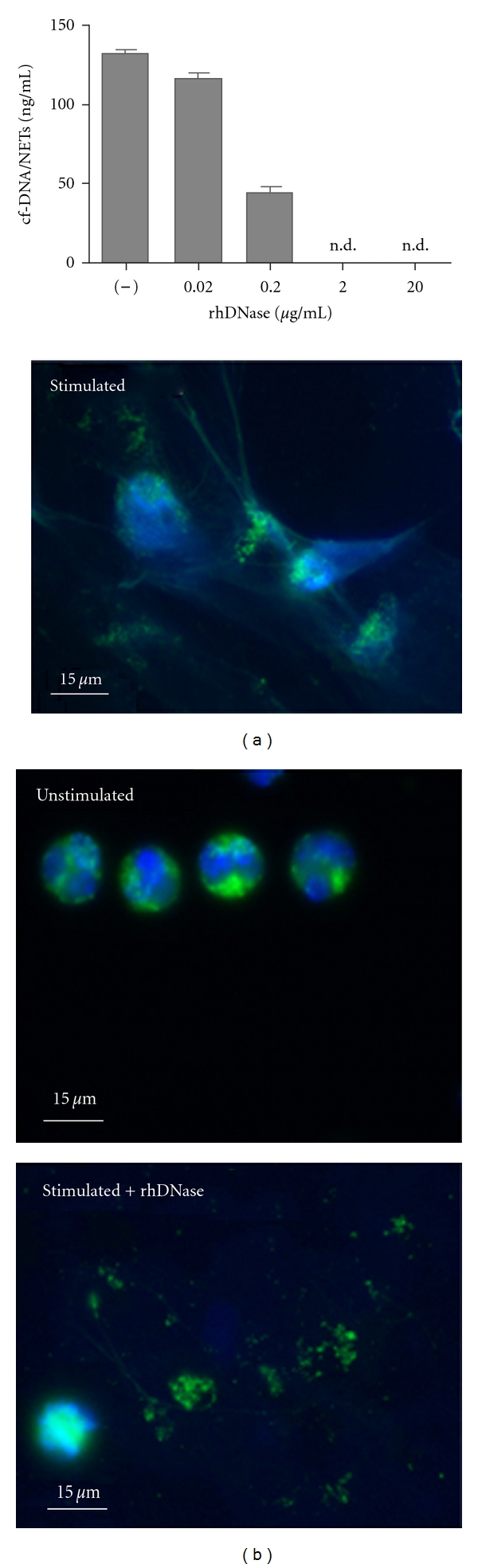
Degradation of NETs by DNase in vitro. (a) Degradation of NETs in a concentration-dependent manner. Neutrophils were isolated from healthy volunteers (*n* = 5) and stimulated 3 h with 50 nM PMA. Then, the supernatants were incubated with 0, 0.02, 0.2, 2.0, and 20.0 *μ*g/mL rhDNase for 30 minutes and values of cfDNA/NETs were determined (n.d.: not detected). Data are presented as mean ± SEM. (b) Immunofluorescence staining of NETs. Neutrophils were stimulated with 30 nM PMA for 3 h and stained for DNA (blue) and MPO (green). Cells were further incubated with 20 *μ*g/mL rhDNase.

In order to prove the ability of DNase to degrade the scaffold of NETs in vitro, supernatants of stimulated neutrophils were incubated with DNase, and NETs were quantified by cf-DNA/NETs assay. As depicted in Figure 4(a), NETs were disintegrated in a concentration-dependent manner. Furthermore, qualitative evidence of DNase-mediated NET degradation has been shown by immunofluorescence (Figure 4(b)). Viable unstimulated neutrophils from healthy volunteers and neutrophils stimulated with PMA were fixed and stained for NET components (blue = DNA, green = MPO). The image of unstimulated neutrophils shows the nuclear localization of DNA and the granular pattern of MPO (top right). After stimulation, morphological changes during NET formation could be determined with loss of nuclear lobules and granular integrity of MPO (left bottom). Exposure of fixed NETs with rhDNase resulted in the disintegration of NETs with loss of DNA structures (right bottom).

## 4. Discussion

Formation of NETs has been discussed as an effective mechanism of the innate immune system and as relevant in infections, sepsis, and autoimmune diseases. NETs have been shown to trap various types of pathogens [21, 24–26]. Although NETs have been demonstrated to effectively enhance bacterial trapping, this antibacterial mechanism occurs at the expense of injury to endothelium, tissue, and organs [21, 27].

We could confirm previously published results that cf-DNA/NETs are enhanced in the serum of trauma patients, especially those who later developed sepsis [28]. In this study we could show that particularly cfDNA/NETs values were enhanced in the very early phase of sepsis or even before clinical manifestation. This suggests a certain regulatory importance of this finding. Furthermore, in this phase DNase values in septic patients were also significantly increased compared to patients without sepsis and healthy volunteers. Moreover, NETs released by stimulated neutrophils in vitro were efficiently degradated by recombinant DNase in a concentration-dependent manner. All the data obtained in this study provide indication for an important pathophysiological role of cf-DNA/NETs and their relationship to DNase in the early phase of sepsis after trauma. The release of DNase may on one hand inhibit the antibacterial effects of NETs on the other hand DNase could protect the tissue from autodestruction caused by inadequate NETs release in septic patients. Furthermore DNase itself may have a regulatory function in NETs formation of neutrophils.

As their structural backbone is composed of chromatin, NETs are destroyed by DNase. The endonuclease DNase is the major nuclease normally produced by the pancreas and salivary glands and is a physiological constituent of human plasma at concentrations of approximately 3 ng/mL [29]. Brinkmann et al. have already shown in vitro that brief treatment of activated neutrophils with DNase abolishes microbial killing by NETs [9]. In our study this DNase-related dissociation of NETs in vitro has been confirmed by immunofluorescence. Furthermore DNase degrades NETs in a concentration-dependent manner. As after trauma DNase levels of all patients correlate to DNase values and both were significantly elevated in the early phase of sepsis after major trauma, an immunological-based interaction is possible. Given that NETs play a role in the pathogenesis of diverse immune disorders, the formation and activity of endogen-released DNases are prerequisites for natural counter regulation. It ought to be taken into account that adequate DNase release may inhibit the antibacterial effects of NETs or protect the tissue from autodestruction in inadequate NETs release in septic patients. Physiologic amounts of NETs are likely to be important in anti-infectious innate immune responses. In contrast, aberrant amounts of NETs, not sufficiently degraded by DNAses within the blood, may occlude capillaries, impair microcirculation, enzymatically damage tissues, and strongly promote inflammation. Patients who have low amounts of DNases in their blood are not able to adequately control NET formation and thus may have a higher risk to develop severe sequelae than patients with normal amounts of DNases.

Beside physiological production of DNases from pancreas and salivary gland cells, some bacteria protect themselves from trapping by degrading the NETs via endogenous DNase [22]. DNases are expressed by many Gram-positive bacterial pathogens [30], but their role in virulence has been unclear until the discovery of NETs. Beiter et al. showed that the surface endonuclease A (EndA) of *Streptococcus pneumoniae *enabled the bacteria to escape the immune system by degrading the DNA scaffolds of NETs [18]. Upon degradation by extracellular DNases excessively accumulated NETs within tissues or capillaries release NET-associated effector molecules enzymes such as neutrophil-derived elastase which may entail severe tissue damage. However, the effects of these proteins when released from NETs after degradation by natural or therapeutic DNase are unclear.

The assay used to label neutrophil-derived NETs by targeting the NET containing cf-DNA directly within serum has been recently developed. It has been shown to be specific for neutrophils since stimulation of other blood cells or other cells did not result in production of NETs or in increased fluorescence signals [22, 31]. The assumption that the cfDNA is a component of floating NETs has been supported by previous studies, in which MPO, as a granule component of NETs, strongly correlates to cfDNA/NETs values [22, 32]. However, as rightly remarked by the reviewer the definite proof in vivo that cfDNA largely derives from hyperactivated neutrophils in patients after multiple trauma has to be done.

## 5. Conclusions

Values of cf-DNA/NETs and DNase are significantly increased in the early phase of sepsis after major trauma. DNase degrades NETs in a concentration-dependent manner and may have a regulatory effect on NET formation in neutrophils. This may inhibit the antibacterial effects of NETs or protect the tissue from autodestruction in inadequate NETs release in septic patients. Furthermore, therapeutic strategies that limit NETs activity, for example, by DNase or DNase inhibitors treatment might prevent neutrophil-derived pathological effects possibly resulting in posttraumatic organ failure.

## Figures and Tables

**Table 1:** Infection site of sepsis and microbiological pathogens.

Subject	Site of infection	Pathogen	Evidence for sepsis, d
1	Pneumoniae	n.d.	8
2	Pneumoniae	*Escherichia coli*	7
3	Pneumoniae	*Klebsiella pneumoniae*	5
4	Pneumoniae	*Klebsiella pneumoniae*	4
5	Pneumoniae	*Morganella morganii*	6
6	Pneumoniae	*Enterobacter cloacae*	4
7	Soft tissue	*Enterobacter cloacae*	8
8	Pneumoniae	*Morganella morganii*	4
		*Klebsiella oxytoca*	
9	Pneumoniae	*Escherichia coli*	7
10	Pneumoniae	*Klebsiella pneumoniae*	7
11	Pneumoniae	*Klebsiella oxytoca*	4
12	Pneumoniae	*Klebsiella pneumoniae*	4
13	Pneumoniae	*Enterococcus faecalis*	5
	Soft tissue	*Enterococcus faecalis*	
14	Pneumoniae	*Enterobacter cloacae*	8

d indicates days after trauma, n.d.: not determined.
